# Gender-specific differences in cannibalism between a laboratory strain and a field strain of a predatory mite

**DOI:** 10.1007/s10493-018-0232-4

**Published:** 2018-02-22

**Authors:** A. M. Revynthi, A. Janssen, M. Egas

**Affiliations:** Institute of Biodiversity and Ecosystem Dynamics, University of Amsterdam, P.O. Box 94248, 1090 GE Amsterdam, The Netherlands

**Keywords:** Haplodiploidy, Relatedness, *Phytoseiulus persimilis*, Acari

## Abstract

Many phytoseiid species, including *Phytoseiulus persimilis*, are known to engage in cannibalism when food is scarce and when there is no possibility to disperse. In nature adult females of *P. persimilis* are known to disperse when prey is locally depleted. Males, in contrast, are expected to stay and wait for potential mates to mature. During this phase, males can obtain food by cannibalizing. Therefore, we hypothesize that male *P. persimilis* exhibit a higher tendency to cannibalize than females. Because rearing conditions in the laboratory usually prevent dispersal, prolonged culturing may also affect cannibalistic behavior. We hypothesize that this should especially affect cannibalism by females, because they consume far more food. We tested these hypotheses by comparing males and females from two strains, one of which had been in culture for over 20 years, whereas the other was recently collected from the field. It is known that this predator can discriminate between kin and non-kin and prefers cannibalizing the latter, hence to construct lines with high relatedness we created isofemale lines of these two original strains. We subsequently tested to what extent the adult females and males of the original strains and the isofemale lines cannibalized conspecific larvae from the same strain/line in a closed system. Relatedness with the victims did not affect cannibalistic behavior, but males engaged more often in cannibalism than females, and females of the laboratory strain engaged more in cannibalism than those of the field strain, both in agreement with our ideas. We hypothesize that the difference in cannibalism between the two genders will increase when they have the alternative to disperse.

## Introduction

Cannibalism, the act of killing and consuming an individual of the same species, is a common phenomenon across the animal kingdom. It occurs in various taxa such as birds (Cain et al. 1984), fish (Okuda and Yanagisawa 1996), frogs (Ringler et al. 2017), salamanders (Takatsu and Kishida 2015), spiders (Bilde and Lubin 2001), insects (Tschinkel 1981) and mites (Yao and Chant 1989). Animals prey on conspecifics mainly as a response to low food densities (Fox 1975), but cannibalism is also affected by stress, kin competition or mate competition and prey vulnerability (Fox 1975; Polis 1981; Pfennig 1997). Cannibalism can act as a life boat mechanism and result in species persistence when food is scarce (van den Bosch et al. 1988). On the down side, cannibalism can result in injuries, pathogen transmission and, in case the victim is a relative, in loss of inclusive fitness (Pfennig 1997).

Many mite species of the Phytoseiidae family are known for their cannibalistic behavior, and the cannibalistic stage commonly used in experiments is the gravid adult female feeding on juveniles (Schausberger 2003). Females are often predicted to cannibalize more than males, because they use more resources and require more protein for egg production; reproduction is energetically costly (Harshman and Zera 2007). Many of the experiments on cannibalism in phytoseiids are done in closed arenas, where cannibal and victim cannot escape. Hence, these experiments test for the possibility of cannibalism to occur rather than assessing its importance under natural conditions where individuals can disperse. For phytoseiids, this is essential because typical populations of predators and prey are short-lived, and end either by the host plant of the prey being overexploited or by the prey being exterminated by the predators (Janssen and Sabelis 1992; Pels and Sabelis 1999). At the end of this interaction period, the predators disperse in search of new prey patches. Adult females disperse earlier than adult males and juvenile stages (Pels 2001); once inseminated, females need food to reproduce, and juveniles should become mature and mate before dispersal. Consequently, males may delay their dispersal, waiting for new mates to develop. In order to survive, males and juveniles can engage in cannibalism. In previous experiments with predatory mites, we indeed observed much cannibalism by adult males on larvae under conditions of low prey densities (Revynthi et al. submitted). Hence, we postulate that males may have a higher tendency to cannibalize than females.

It is generally accepted that culturing organisms may result in changes in behavior, life history and genetic variation (Mackauer 1976; Hopper et al. 1993), and these changes may affect cannibalism (Dennehy et al. 2001). When rearing phytoseiids, dispersal is often prevented by lethal (water) barriers to keep the mites contained. However, this results in strong selection against dispersal behavior because individuals that try to disperse either end up dead in some barrier, or lose time and possibly energy when attempting to escape. It has been suggested that under conditions of laboratory cultures, higher cannibalism tendency is unintentionally selected for: due to space limitation, predators cannot disperse in search of more food when the prey are temporarily eliminated (Elliot et al. 2002). Instead, in response to the low food conditions, they may prey on their conspecifics. This would suggest that laboratory strains have a higher tendency to cannibalize than strains in the field (where the predators can disperse), and this would hold especially for adult females as they need more food in order to produce offspring.

However, rearing for longer periods may also increase the relatedness among individuals, and it is known that phytoseiids tend to avoid kin-cannibalism (Schausberger and Croft 2001). This may result in lower rates of cannibalism in strains that have been in culture for a longer period. For strains in the field we hypothesize that there is a bigger effect of relatedness on cannibalism rate: higher when cannibalizing among population members, low when cannibalizing among family members.

In the present study we have tested whether a recently collected strain of the predatory mite *Phytoseiulus persimilis* is more or less cannibalistic than a strain that has been in culture for more than 20 years. Several studies investigated the cannibalistic behavior of adult females of this species (Yao and Chant 1989; Walzer and Schausberger 1999; Schausberger and Croft 2001; Schausberger 2007; Schausberger and Hoffmann 2008), but not cannibalism by adult males. To test our hypotheses that males cannibalize more than females, we investigated cannibalism in both males and females and tested whether males and females of both strains differed in the tendency to cannibalize among members of the same strain and among members of the same family.

## Materials and methods

### Plant and prey cultures

Lima beans (*Phaseolus lunatus*) were used as a host and were grown from seeds in a climate room (25 °C, 60% RH, 16L:8D) free of herbivores. The spider mites (*Tetranychus urticae*) that were used as food for *P. persimilis* were originally collected from cucumber plants in a commercial greenhouse in May 1994 (Pallini et al. 1997). They were reared on lima bean plants in a walk-in climate room (26 °C, 60% RH, 16:8/L:D).

### Predatory mites

Two strains of *P. persimilis* were used. One strain derived from Koppert Biological Systems (Berkel en Roderijs, The Netherlands) in 1994 and one from Alcamo in Sicily in 2014 (see Revynthi et al. submitted) have since been reared in our laboratory under identical conditions. These two strains were chosen because we have information about their dispersal behavior (Pels and Sabelis 1999; Revynthi et al. submitted) and because we were interested in observing whether there were differences in the cannibalistic behavior between a laboratory strain, i.e., Koppert, and a strain recently collected from the field, i.e., Alcamo. The predators were kept in closed rearing cages, which allowed the predators to leave and subsequently return to the prey patch (as described in Pels and Sabelis 1999) inside a climate room at 25 °C, 70% RH and 16L:8D photoperiod. To test for an effect of kinship, an isofemale line of each strain was created by isolating a gravid female of *P. persimilis* from the culture and introducing it individually in a separate rearing unit with prey (*T. urticae*). The female and her offspring were allowed to reproduce and create a family for at least 3 months before the start of the experiments. *Phytoseiulus persimilis* has a generation time of 7 days at 25 °C (Laing 1968; Sabelis 1981), therefore the period of 3 months results at least in 12 generations of sib-mating. The strains and isofemale lines were fed 3× per week by introducing two bean leaves (*P. lunatus*) infested with spider mites (*T. urticae*).

To obtain sufficient numbers of gravid females and males of the same age (2-day-old adults), as well as larvae, cohorts were created as follows. Ten gravid female predatory mites from each of the two strains and the two isofemale lines were placed on a spider mite-infested bean leaf on a bed of water-saturated cotton wool in a Petri dish (14 cm diameter, 2 cm high). In this way, the leaves remained turgid for at least 10 days. The gravid females were allowed to oviposit for 48 h, after which they were removed and only their eggs and prey were left on the leaves. The cohorts were kept in the same climate room as the cultures.

### Evaluation of cannibalistic behavior

To measure the cannibalistic tendency of the predatory mites in a closed system that did not allow for dispersal, small plastic cups (2.8 cm diameter, 2.2 cm high) were used. Each cup had a lid with a hole (12 mm diameter) covered with mite-proof gauze (80 μm mesh) for ventilation. 48 h prior to the start of the experiment, males and gravid females from the cohorts described above were individually isolated in a cup, which contained a bean leaf disc (24 mm diameter) on water-saturated cotton wool. The predators did not receive any food, hence were starved at the end of this period (48 h).

At the start of the experiment, three young predator larvae from the same culture as the adult and from a cohort started 3 days earlier were transferred to a cup similar to those described above. Subsequently, a starved adult predatory male or female was released in the same cup; hence, adults and larvae originated from the same culture, but were produced in separate cohorts. The predator was observed 5 min after its release and subsequently every 15 min for a total period of 1 h. Every time the number of alive and consumed larvae was recorded. Cannibalized larvae were recognized by the carcass from which the hemolymph was removed (Yao and Chant 1989). Replicates where a larva had molted to protonymph or died from natural causes were excluded. There were eight treatments, each with 30 replicates: non-kin female Koppert, non-kin male Koppert, non-kin female Alcamo, non-kin male Alcamo, kin female Koppert, kin male Koppert, kin female Alcamo and kin male Alcamo. In the ‘non-kin’ treatment, predators and larvae came from different cohorts of the same main culture; in the ‘kin’ treatment, predators and larvae were from different cohorts of the same isofemale line. The experiment was conducted in 12 blocks (days) and each block contained all the treatments.

### Statistical analysis

To estimate which of the two genders and/or strains engaged more often in cannibalism and whether kinship affected their behavior a generalized linear mixed effect model with a binomial error distribution was used (GLMER of the lme4 package; Bates et al. 2015). Because on average only one cannibalistic event occurred during the observation period, we analyzed the data as the occurrence of cannibalism. Thus, the response variable was a binomial variable (cannibalism or not), gender, strain, kinship and their interactions were the fixed factors and block was used as a random factor. The analysis was performed using R v.3.3.3 (R Development Core Team 2017).

## Results

**Fig. 1:**
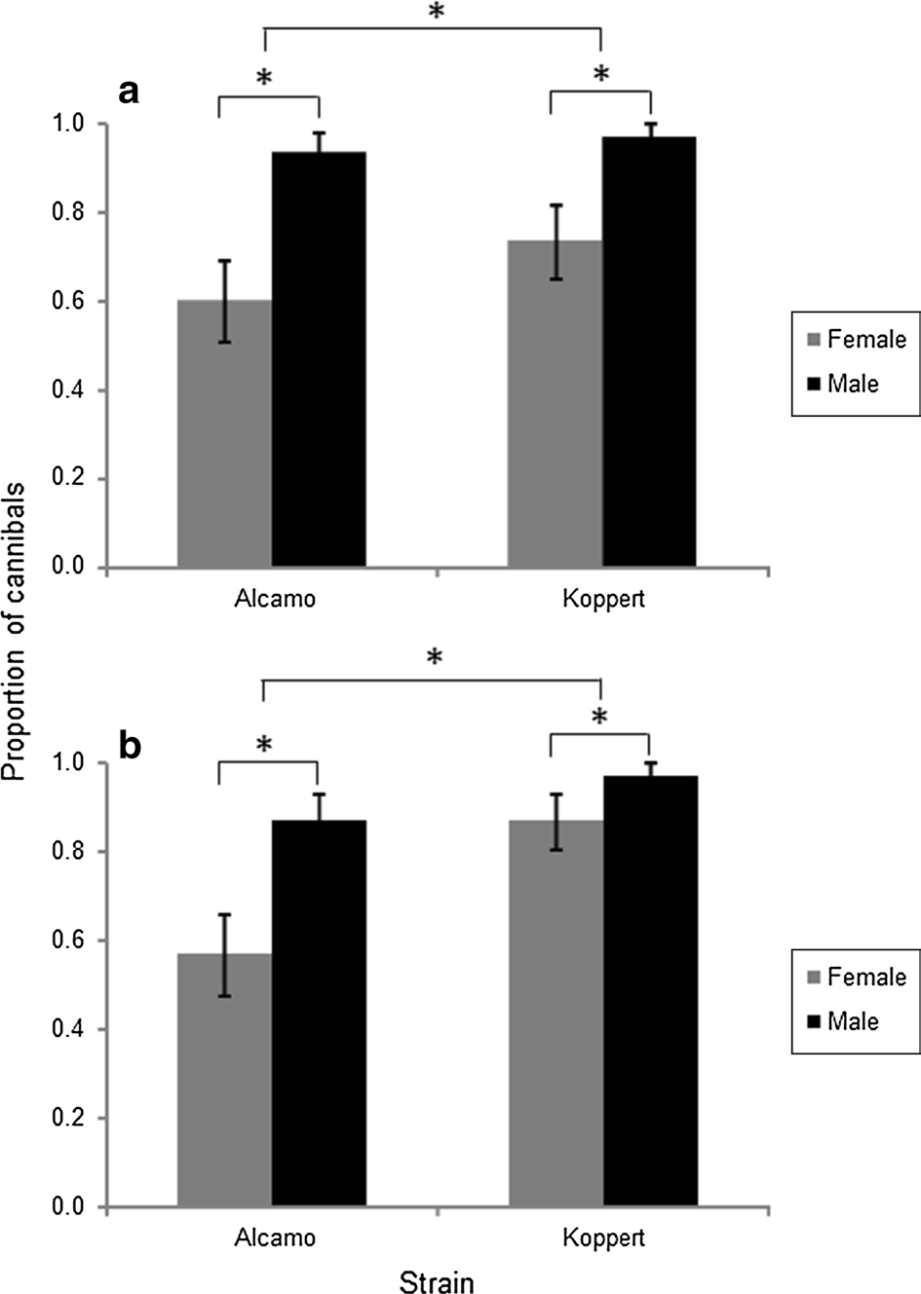
Mean (± SE) proportions of adult males (black bars) and females (grey bars) of two strains (Alcamo and Koppert) of *Phytoseiulus persimilis* that cannibalized on related larvae (kin; panel **a**) or unrelated larvae (non-kin; panel **b**). Asterisks indicate significant differences. N = 30 for each bar

Male predators engaged in cannibalism more often than females (Fig. 1; GLMER: χ^2^ = 25.5, *df* = 1, *P* < 0.0001). The Koppert strain had more cannibals than the Alcamo strain (GLMER: χ^2^ = 8.93, *df* = 1, *P* = 0.003). This was because females of the Koppert strain cannibalized more than females of the Alcamo strain (GLMER, χ^2^ = 6.71, *df* = 1, *P* = 0.0096), but cannibalism by males was not different (χ^2^ = 2.24, *df* = 1, *P* = 0.14). Kinship did not affect cannibalistic behavior (GLMER: χ^2^ = 0.032, *df* = 1, *P* = 0.86). None of the interactions of factors was significant (all *P* > 0.20).

## Discussion

We found that a laboratory strain (Koppert) had a higher tendency to cannibalize than a field strain (Alcamo), confirming the suggestion that higher cannibalism tendency is unintentionally selected for under conditions of laboratory cultures (Dennehy et al. 2001; Elliot et al. 2002). Our results, however, show high levels of cannibalism in a field strain. Moreover, the difference in cannibalistic tendency between the strains was small.

Earlier studies have focused on the voracity of cannibalistic females of *P. persimilis* (Walzer and Schausberger 1999) and their ability to discriminate between kin and non-kin (Schausberger and Croft 2001). To the best of our knowledge, this is the first time that the cannibalistic behavior of male predators of *P. persimilis* was investigated showing that the males of two strains were more prone to cannibalize than gravid females, regardless of relatedness with the victims.

Predatory mites that had been in culture for a long period had a significantly higher tendency to cannibalize than a recently collected strain of the same species. Interestingly, males cannibalized significantly more than females. This difference is probably caused by differences in the behavior of males and females: whereas females search for prey, males search for conspecifics to mate. However, there is an alternative explanation for the differences in the cannibalistic behavior of males and females, which is based on asymmetries in relatedness with offspring. Inclusive fitness theory (Hamilton 1964a, b; Gardner et al. 2011) suggests that evolution will favor the individuals that are able to recognize relatives and avoid cannibalizing them, regardless the gender (Pfennig 1997). In haplodiploid systems, however, differences are expected between the genders due to the difference in relatedness with the victim between male and female cannibals. Phytoseiid mites are pseudo-arrhenotokous (Schulten 1985): males and females both derive from fertilized eggs but only the females remain diploid and carry both maternal and paternal chromosomes (McMurtry et al. 1970). Males lose the paternal set of chromosomes shortly after syngamy (Helle et al. 1978; Sabelis and Nagelkerke 1988). Hence, in these predators, the adult female is expected to suffer a fitness cost from cannibalizing any of her offspring because each offspring carries one set of her genes, whereas the adult male only contributes genetically to daughters, and therefore does not suffer from cannibalizing on sons of his mate. Thus it can be hypothesized that gravid adult females are less prone to cannibalism than males, despite their higher energy requirements. We also expect that males would specifically cannibalize other, immature males, because this reduces future competition for mates, and by letting female immatures live, they increase the chance on a future mate. This remains to be tested.

We found no effect of kinship on cannibalism, suggesting that when cannibals do not have a choice between kin and non-kin victims, the decision to cannibalize is not affected by the degree of relatedness with the potential victims. Hence, this suggests that no inbreeding effects occurred in the strain that had a long history of being cultured or that inbreeding did not affect the tendency to cannibalize. To our best knowledge, inbreeding effects in *P. persimilis* are absent unless long periods of strong inbreeding are invoked (Poe and Enns 1970). This is common for haplodiploid species, where selection against recessive alleles always acts on the haploid males and the frequency of such alleles therefore remains low, except for genes that specifically code for female traits such as egg production (Tien et al. 2015). We also did not observe population declines or any other adverse effects of inbreeding in either of the two isofemale lines. The isofemale lines were started with one female of each strain. This female was therefore potentially not representative for the entire population. For example, it could have had a higher genetic tendency for cannibalism. This would then have resulted in less variation in the cannibalistic behavior in the isofemale lines than in the original lines, because those mites are genetically fixed whereas the original lines were genetically more diverse. However, we found no such difference in variation in the cannibalistic behavior between strains and lines.

Several studies have focused on kin recognition in phytoseiid mites (Faraji et al. 2000; Schausberger and Croft 2001), but also in other animal taxa (Pfennig 1997; Bilde and Lubin 2001; Parsons et al. 2013; Bayoumy and Michaud 2015; Ringler et al. 2017). In the spider species *Stegodyphus lineatus* cannibalism was lower in groups where all the individuals were kin than in mixed groups of kin and non-kin (Bilde and Lubin 2001). Bayoumy and Michaud (2015) showed that females of the Coleoptera species *Hippodamia convergens* discriminate between filial and non-filial egg clusters and preferentially cannibalize the latter. Schausberger and Croft (2001) show that *P. persimilis* is able to discriminate between kin and non-kin larvae and preferred to cannibalize the latter. Even though our study was not focused on kin discrimination of *P. persimilis*, we explored whether the level of relatedness with the prey could have affected cannibalistic behavior. The lack of variation between the kin and non-kin treatment does not contradict earlier reports of kin discrimination in this species (Schausberger and Croft 2001; Schausberger 2004) because we did not offer the cannibals a choice between kin and non-kin victims.

In natural settings, the predators can opt out of cannibalizing by dispersing away from the patch without prey and search for a new prey patch. For the two strains used here, we have information about their dispersal behavior (Pels and Sabelis 1999; Revynthi et al. submitted). In wind tunnel experiments, both strains showed a tendency to disperse only after heterospecific prey were depleted, showing the so-called ‘killer’ strategy of prey exploitation (van Baalen and Sabelis 1995). Theoretical work on the evolution of cannibalism and predator dispersal predicts that predators with the ‘killer’ strategy are selected for higher cannibalistic tendency (Pels 2001). Given the variation for this prey exploitation behavior (Pels and Sabelis 1999; Revynthi et al. submitted), we argue that there may also be variation in cannibalistic tendency among natural populations of *P. persimilis*. Future research should explore whether the two genders will show similar behavior as observed in this study when they have the option to disperse.
